# Evidencing an inner-sphere mechanism for NHC-Au(I)-catalyzed carbene-transfer reactions from ethyl diazoacetate

**DOI:** 10.3762/bjoc.11.245

**Published:** 2015-11-20

**Authors:** Manuel R Fructos, Juan Urbano, M Mar Díaz-Requejo, Pedro J Pérez

**Affiliations:** 1 Laboratorio de Catálisis Homogénea, Unidad Asociada al CSIC, CIQSO-Centro de Investigación en Química Sostenible and Departamento de Química, Universidad de Huelva, Campus de El Carmen, 21007 Huelva, Spain

**Keywords:** carbene transfer, inner sphere, gold catalysis, O–H functionalization, olefin cyclopropanation

## Abstract

Kinetic experiments based on the measurement of nitrogen evolution in the reaction of ethyl diazoacetate (N_2_CHCO_2_Et, EDA) and styrene or methanol catalyzed by the [IPrAu]^+^ core (IPr = 1,3-bis(diisopropylphenyl)imidazole-2-ylidene) have provided evidence that the transfer of the carbene group CHCO_2_Et to the substrate (styrene or methanol) takes place in the coordination sphere of Au(I) by means of an inner-sphere mechanism, in contrast to the generally accepted proposal of outer-sphere mechanisms for Au(I)-catalyzed reactions.

## Introduction

**Scheme 1:**
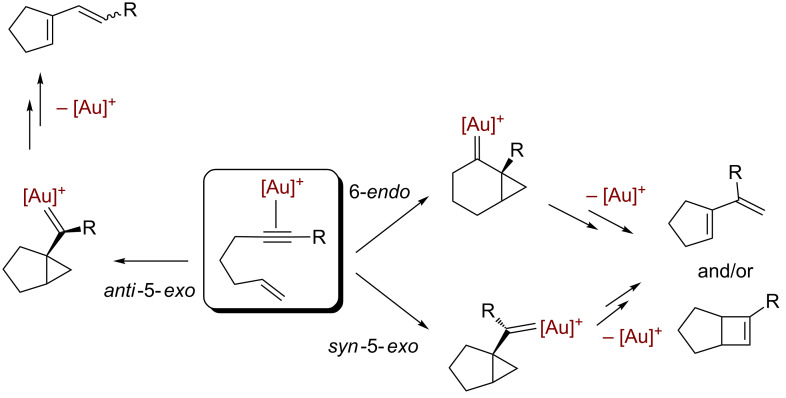
The Au(I)-catalyzed skeletal rearrangement of the [2 + 2] cycloaddition of 1,6-enynes that involves gold–carbene intermediates.

The discovery of the catalytic capabilities of soluble gold(I) species toward the hydration of alkynes by Teles and co-workers [1] is considered as the rising of the golden era for the use of this metal in homogeneous catalysis [2–3]. A number of transformations have been reported to date [4–13], most of them based on a particular feature of gold: a singular carbophilicity that enhances the electrophilic activation of multiple bonds upon coordination followed by subsequent inter- or intramolecular reaction with nucleophiles. Most of the reported systems contain an unsaturated fragment that is activated upon coordination to the gold center, thus triggering further transformations, the formation of very reactive gold–carbene intermediates being proposed [4–14]. As a representative example, the skeletal rearrangement of the [2 + 2] cycloaddition of 1,6-enynes [4] is shown in Scheme 1, where three different gold–carbene intermediates are involved in the possible transformations.

**Scheme 2:**
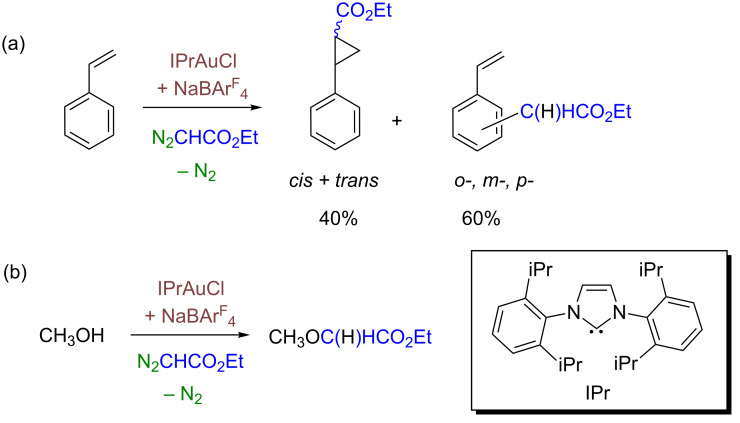
The catalytic activity of IPrAuCl + NaBAr^F^_4_ in the carbene-transfer reaction to styrene or methanol.

A different reaction in which the formation of gold–carbene intermediates has been proposed arises from the interaction of a gold(I) source and a diazo compound. It was not until 2005 that the first example of this transformation was reported by our group [15–16], when the complex IPrAuCl (**1**) (IPr = 1,3-bis(diisopropylphenyl)imidazole-2-ylidene), in the presence of NaBAr^F^_4_ (BAr^F^_4_^−^ = tetrakis(3,5-bis(trifluoromethyl)phenyl)borate) as a halide scavenger, induced the incorporation of the :CHCO_2_Et group from N_2_CHCO_2_Et to styrene (Scheme 2a) or methanol (Scheme 2b), among others. With the former, in addition to the formation of the expected cyclopropanes, a second type of product was observed, derived from the incorporation of the carbene :CHCO_2_Et unit to the C(sp^2^)–H bonds. For methanol as the reactant, the ether derived from the functionalization of the O–H bond was obtained.

**Scheme 3:**
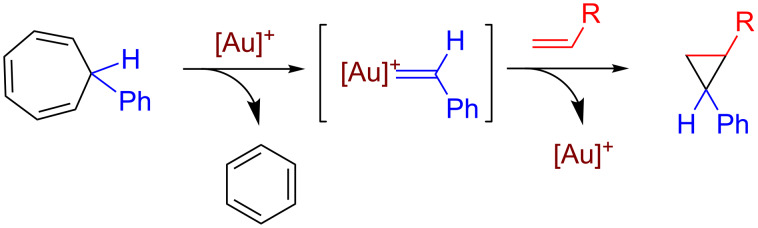
The gold-promoted decarbenation reaction described by Echavarren and co-workers.

A transformation related to this contribution is the reaction published by Echavarren (Scheme 3). In this reaction, the complex [LAu(NCR)]SbF_6_ (L = tertiary phosphine ligand) abstracted the carbene group :CHPh from a tropilium derivative, and further transferred the gold-bonded carbene group to an olefin [17–18]. This contribution constituted a breakthrough in gold-mediated carbene-transfer reactions since the carbene source lacks of the well-known instability of some of the diazo reagents.

**Scheme 4:**
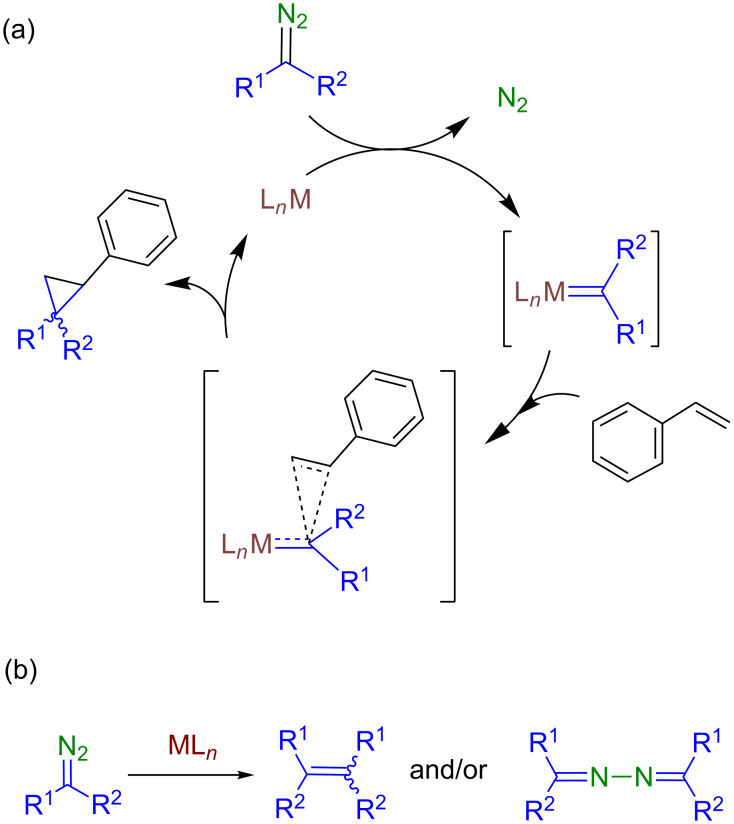
(a) General representation of the metal-catalyzed carbene-transfer reaction (olefin cyclopropanation). (b) Side-reactions of carbene or diazo coupling commonly observed.

The reactions shown in Scheme 2 and Scheme 3 have been explained through the appearance of a [LAu=CR^1^R^2^]^+^ intermediate, not detected nor isolated, that further reacts with a non-coordinated nucleophile (i.e., by means of an outer-sphere mechanism). Those intermediates are quite reactive, in contrast to similar but low reactive gold–carbene complexes recently and independently described by the groups of Fürstner [19] and Straub [20]. Scheme 4a shows a generally accepted mechanism for the metal-catalyzed carbene transfer from diazo compounds to nucleophiles [21–27], where the formation of C–C or C–X bonds takes place throughout a transition state in which no interaction of the substrate and the metal center exists (styrene is shown as an example). In many cases, a non-desired side reaction is also observed, in which two molecules of the diazo compound convert into an olefin and/or an azine (Scheme 4b), the second acting as a nucleophile attacking the metallocarbene intermediate [28]. However, our previous studies have shown that this gold-based catalytic system does not induce such non-desired carbene homocoupling [15–16].

In this contribution we report the results obtained from a kinetic study carried out with the IPrAuCl complex that allows proposing a plausible inner-sphere mechanism in which the substrate (styrene or methanol) seems to remain coordinated to the gold center, a feature that could be extended to many of the reported catalytic systems involving Au(I) complexes.

## Results and Discussion

### The probe reactions

**Figure 1:**
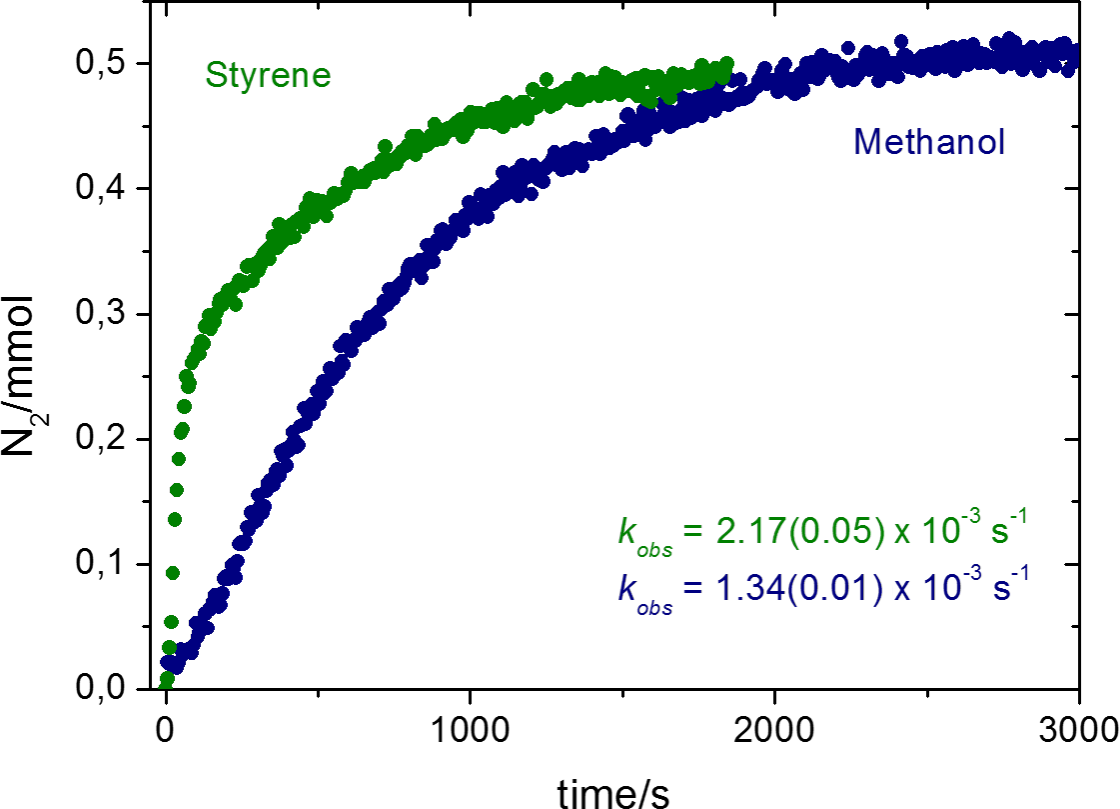
Plot of evolved nitrogen with time for the reactions of EDA with styrene or methanol.

As mentioned above, we have already described the potential of the system IPrAuCl + NaBAr^F^_4_ to promote the catalytic decomposition of ethyl diazoacetate (N_2_CHCO_2_Et, EDA) and functionalize styrene or methanol (see Scheme 2). Since molecular nitrogen is evolved in these transformations, we have monitored the pressure above the reaction mixtures of EDA and styrene or methanol in the presence of catalytic amounts (5 mol %) of IPrAuCl + NaBAr^F^_4_. It is worth noting that the catalyst precursors were dissolved in the neat substrate (5 mL) and stirred for 30 min to ensure halide abstraction prior to EDA addition. The experiments have been carried out using a flask connected to a pressure gauge that provides the variation in the increase of the internal pressure (see Experimental). Figure 1 shows the plots of the N_2_ concentration (mmol) vs time, from which *k*_obs_ for N_2_ liberation have been obtained as 2.17(0.05) × 10^−3^ s^−1^ and 1.34(0.01) × 10^−3^ s^−1^ for styrene and methanol, respectively. Thus, both reactions decomposed EDA with similar rates within the same order of magnitude. Given the experimental fact that this catalytic system does not promote the carbene coupling side reaction, the amount of measured N_2_ exclusively corresponds to that evolved from the formation of the gold–carbene intermediate in the path to the products.

### The effect of substrate concentration

**Figure 2:**
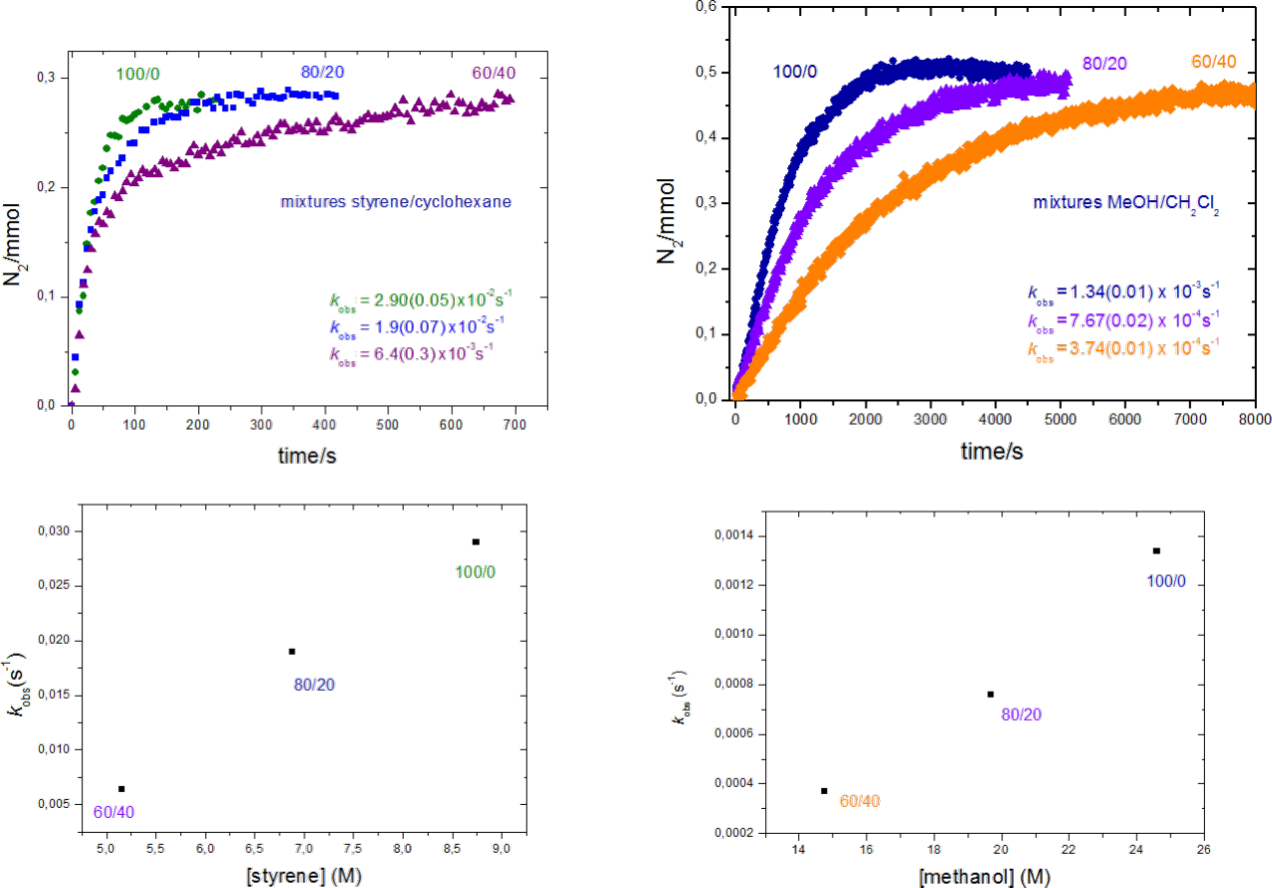
Top: Plots of evolved nitrogen with time for the reactions of EDA with styrene (left) or methanol (right) varying the relative substrate:catalyst ratio, at a constant catalyst value. Bottom: Plots of *k*_obs_ vs substrate concentration showing a direct correlation between both magnitudes.

A second set of experiments has been performed varying the catalyst to substrate ratio, but maintaining the concentration of the catalyst as a constant. Thus, 100:0, 80:20 and 60:40 v/v mixtures of styrene and cyclohexane, respectively, have been reacted with EDA (0.285 mmol) with the same catalyst precursor (see Experimental). Under these conditions, cyclohexane is much less reactive than styrene and acts as an inert solvent [29]. The kinetic curves for these three experiments are shown in Figure 2. The plot of *k*_obs_ vs substrate concentration evidences a direct correlation between styrene concentration and *k*_obs_. The same effect is observed in an array of experiments carried out with mixtures of methanol and methylene chloride (Figure 2). These results unambiguously indicate that the release of nitrogen takes place at a faster rate when increasing the amount of the reactant, styrene or methanol.

It is worth mentioning that the addition of 5 equiv of NaBAr^F^_4_ did not induce any change in the reaction rate compared with that of one equiv, assessing that the halide-free catalytically-active gold species is available in the latter case. Also, the fact that the same behavior regarding the “dilution effect” shown in Figure 2 is observed with cyclohexane or dichloromethane as dilution agents must be interpreted as the result of their independent behavior in the process, not being involved in any effect relative to polarity or solubility issues.

### Mechanistic interpretation

It has been reported by several authors that the use of LAuX (X = halide) as catalyst precursor requires the addition of a halide scavenger with a weekly coordinating ligand (such as NTf_2_, BAr^F^_4_, SbF_6_) [30–31]. Cationic complexes of type [LAu(NCR)]^+^ have also been isolated previously [32–33]. One way or the other, the incorporation of the reactant has been proposed to be associative in most cases, the reaction then being triggered from there, usually through an outer-sphere mechanism. To the best of our knowledge, there are no evidences which support the alternative route, the inner-sphere mechanism.

**Scheme 5:**
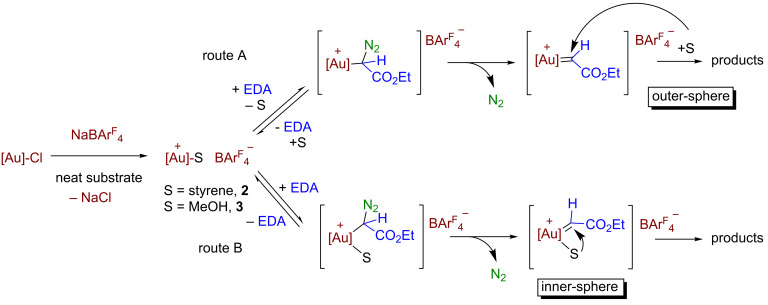
The outer- and inner-sphere routes for this transformation.

The kinetic data available from the previous section have provided the following statements: (i) there is only a slight effect of the nature of the substrate, styrene or methanol, in the rate of evolution of N_2_, and (ii) the reaction rate is affected by the relative substrate:catalyst ratio, both magnitudes being directly correlated. Scheme 5 displays a feasible catalytic cycle for these transformations. The mixture of IPrAuCl and NaBAr^F^_4_ in styrene or methanol ensures the formation of the cationic species [IPrAu(sty)]BAr^F^_4_ (**2**) or [IPrAu(MeOH)]BAr^F^_4_ (**3**) similar to structurally characterized [IPrAu(NCMe)]BF_4_ [32–33]. The addition of EDA originates the immediate evolution of N_2_, the gold–carbene intermediate must form from **2** or **3**. Also, this must be the rate determining step, efforts to detect such intermediates having proven unsuccessful. The commonly accepted pathway (route A, Scheme 5) would suppose an exchange of the substrate ligand with the diazo compound and then evolution of nitrogen. However, that first step of substrate dissociation would be in disagreement with the observation of the enhancement of the reaction rate of nitrogen evolution when increasing the substrate:catalyst ratio, the dissociation of the substrate should be disfavoured at larger substrate concentration. Thus, there is only a plausible explanation for the experimental data (route B, Scheme 5): the substrate remains coordinated while the diazo compound coordinates and eliminates nitrogen, the carbene transfer taking place between two ligands, the carbene and the substrate. This approach would also explain the lack of formation of the olefins derived from carbene coupling with this Au(I)-based system.

## Conclusion

The experimental results obtained from the measurement of N_2_ evolution in [IPrAu]^+^-catalyzed carbene transfer from ethyl diazoacetate have allowed proposing that both the carbene and the substrate (styrene or methanol) are bonded to the Au(I) center previously to the corresponding coupling, that is, the functionalization reaction takes place throughout an inner-sphere mechanism. This is not the commonly proposed mechanism for most of the different Au(I)-catalyzed reactions, for which an outer-sphere mechanism has been frequently assumed. We hope that the findings reported herein could be verified for other Au(I)-catalyzed transformations.

## Experimental

### General methods

All preparations and manipulations were carried out under an oxygen-free nitrogen atmosphere using conventional Schlenk techniques. Solvents were rigorously dried previously to their use. The substrates were purchased from Aldrich. The complex IPrAuCl and NaBAr^F^_4_ were prepared according to the literature [34–35]. NMR spectra were performed on Agilent 400 MR and 500 DD2 spectrometers. GC data were collected with a Varian GC-3900 spectrometer with a FID detector.

### Kinetic experiments

**Figure 3:**
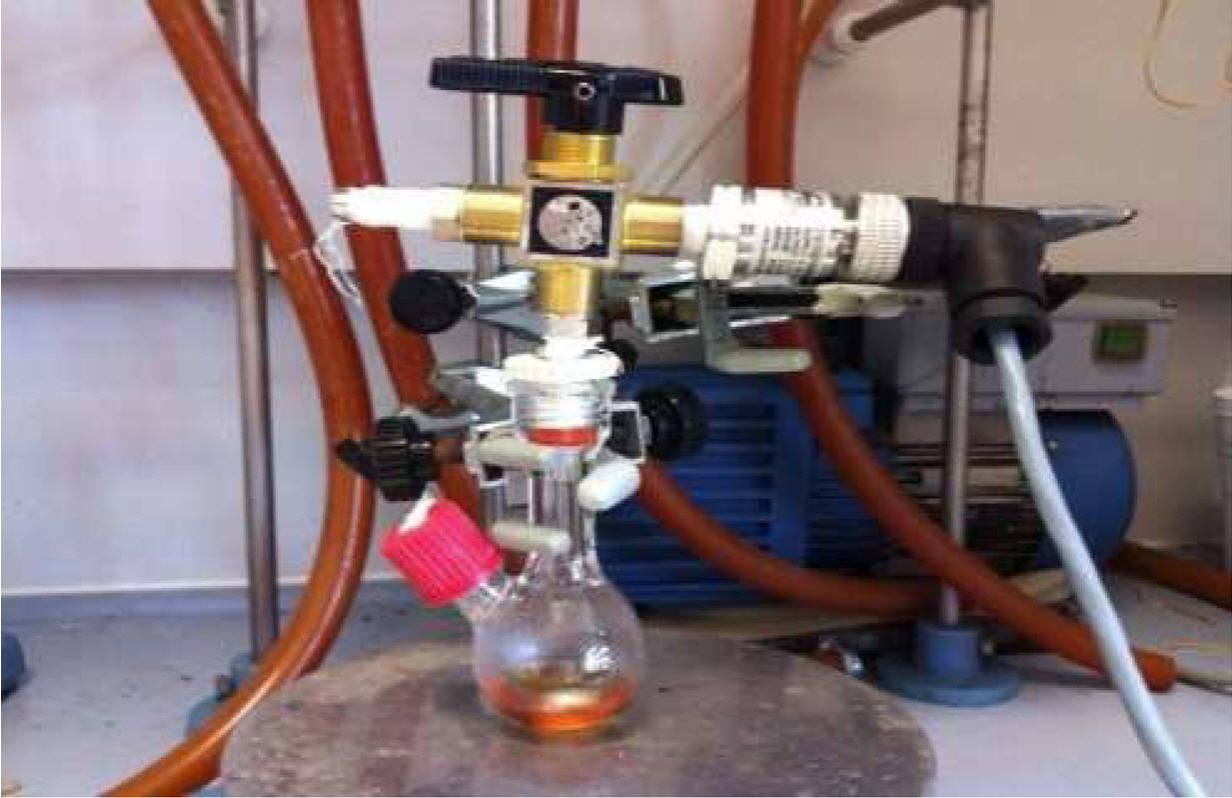
The experimental device for the measurement of N_2_ evolution.

The kinetic experiments have been carried out similarly to previous procedures from our laboratory [36]. For the sake of clarity, the methodology is briefly described herein. A ManontheMoonTech X201 (http://www.manonthemoontech.com) device consisting of a stainless-steel gas reservoir doubly connected to a pressure transmitter has been employed for the measurement of nitrogen evolution. The variation of the inner pressure inside the reaction flask (Figure 3) is measured with an electronic pressure meter/controller (EL-Press,Bronkhorst HI-TEC).

In a typical experiment, the N_2_ pressure change was measured after the addition of EDA (0.285 mmol) to a stirred solution of substrate (0. 5 mmol), and a mixture of IPrAuCl and NaBAr^F^_4_ (5 mol %) in cyclohexane/CH_2_Cl_2_ (5 mL) at room temperature. *k*_obs_ was obtained from experimental curves upon fitting to an exponential growth equation using ORIGIN software.
